# The *HCN1* p.Ser399Pro variant causes epileptic encephalopathy with super-refractory status epilepticus

**DOI:** 10.1038/s41439-023-00247-8

**Published:** 2023-06-23

**Authors:** Yu Kobayashi, Jun Tohyama, Noriyuki Akasaka, Kei Yamada, Moemi Hojo, Eijun Seki, Masaki Miura, Noriko Soma, Takeshi Ono, Mitsuhiro Kato, Mitsuko Nakashima, Hirotomo Saitsu, Naomichi Matsumoto

**Affiliations:** 1 Department of Child Neurology, National Hospital Organization Nishiniigata Chuo Hospital, Niigata, Japan; 2 Department of Pediatrics, Niigata Prefecture Hamagumi Medical Rehabilitation Center for Disabled Children, Niigata, Japan; 3 Department of Pediatrics, Niigata University Medical and Dental Hospital, Niigata, Japan; 4 Department of Pediatrics, Showa University School of Medicine, Tokyo, Japan; 5 Department of Biochemistry, Hamamatsu University School of Medicine, Hamamatsu, Japan; 6 Department of Human Genetics, Yokohama City University Graduate School of Medicine, Yokohama, Japan

**Keywords:** Disease genetics, Genetic counselling

## Abstract

*HCN1* is one of four genes encoding hyperpolarization-activated cyclic nucleotide-gated channels. The phenotypic spectrum associated with *HCN1* variants ranges from neonatal developmental and epileptic encephalopathy to idiopathic generalized epilepsy. We report a Japanese patient with repetitive focal seizures and super-refractory status epilepticus since early infancy caused by a *de novo HCN1* variant, NM_021072.4, c.1195T>C, p.(Ser399Pro). This variant might have a dominant-negative effect on channel function, leading to severe epileptic encephalopathy.

Hyperpolarization-activated cyclic nucleotide-gated (HCN) channels mediate a cationic current that stabilizes the neuronal membrane potential against excitatory or inhibitory input and regulates neuronal network excitability^1,2^. *HCN1* (NM_021072), one of the four genes that encode HCN channels^1^, is highly expressed in the neocortex, hippocampus, and brainstem of the central nervous system^3^. It was therefore presumed that pathogenic *HCN1* variants could produce pharmacoresponsive epilepsy or developmental and epileptic encephalopathy (DEE). In 2014, Nava *et al*. identified five different *de novo HCN1* variants in patients with Dravet-like syndrome without *SCN1A* and *PCDH19* variants or fever-sensitive epileptic encephalopathy^4^. Additionally, recent studies have revealed that the phenotypic spectrum associated with *HCN1* variants ranges from genetic epilepsy with febrile seizures plus (OMIM #618482) or genetic generalized epilepsy to neonatal- or infantile-onset DEE^5–10^ (OMIM #615871).

Herein, we report a Japanese patient with a de novo heterozygous *HCN1* variant who had presented since early infancy with repetitive focal seizures and recurrent super-refractory status epilepticus, which were associated with profound developmental delay. This report provides detailed clinical findings that expand the known phenotypic spectrum of *HCN1*-related DEE.

The male patient, the second child of unrelated parents, was born at 39 weeks gestational age after a normal pregnancy and delivery. His birth weight, length, and occipitofrontal circumference were 3380 g (+0.8 standard deviation score [SDS]), 50.8 cm (+0.9 SDS), and 33.8 cm (+0.3 SDS), respectively. His father, elder brother, and maternal uncle had a history of febrile seizures. At 2 months of age, the patient developed convulsive seizures that gradually increased in frequency, occurring in clusters despite administration of phenobarbital and sodium valproate.

He was first admitted to our hospital at 4 months of age. His anthropometric measurements were as follows: body weight 7.6 kg (+0.6 SDS), height 60.5 cm (−1.6 SDS), and occipitofrontal circumference 42.4 cm (+1.3 SDS). Psychomotor development was normal. After admission, right or left hemi-clonic seizures and focal to bilateral tonic–clonic seizures were observed. Shortly afterward, a respiratory infection elicited a high fever and provoked the onset of convulsive status epilepticus. Intravenous anti-seizure medications, including diazepam, midazolam, phenytoin, and lidocaine, were ineffective. Ultimately, thiopental was effective in terminating seizure activity. An interictal electroencephalogram (EEG) showed spike discharges in the left frontal area. An ictal EEG revealed spike-wave discharge with onset in the left fronto-centro-parietal region (Fig. 1A) or right fronto-central region. Laboratory examinations and brain magnetic resonance imaging (MRI) findings showed no abnormalities at that time (Fig. 1B).

**Fig. 1:**
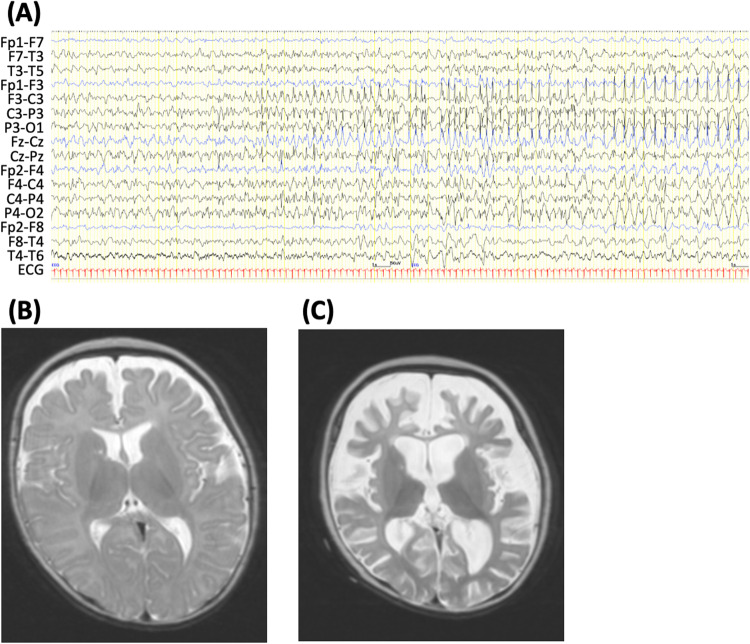
Electroencephalogram (EEG) and axial T2-weighted brain magnetic resonance imaging (MRI) of the patient. **A** An ictal EEG during right hemiclonic seizures at 4 months of age shows spike-wave discharges with onset in the left fronto-centro-parietal region. **B** Brain MRI at 4 months of age, showing normal findings. **C** At 8 months of age, after a second episode of convulsive status epilepticus, brain MRI shows severe frontal-dominant cortical and white matter atrophy with ventricular enlargement.

At 6 months of age, during another respiratory tract infection, the patient developed a prolonged generalized tonic convulsion lasting 30 min, which evolved into repetitive generalized convulsions with prolonged hypoxia. This prolonged seizure was refractory to anticonvulsants, including thiopental, and lasted 36 h. Pentobarbital was given intravenously, and mechanical ventilation was required for 2 weeks. After extubation, seizures were controlled by high-dose phenobarbital treatment combined with potassium bromide and zonisamide. However, the patient’s motor and cognitive development were severely impaired. Brain MRI at 8 months of age showed severe cortical and white matter atrophy (largely in the frontal lobe) and ventricular enlargement (Fig. 1C).

At 10 months of age, brief daily seizures involving grinning or a pale face were noted. At 13 years of age, the patient had generalized hypotonia and spastic quadriplegia with profound intellectual and motor impairment. He could not control his head, spoke no meaningful words and was almost entirely bedridden. An increase in brief seizures with tonic movements elicited the addition of levetiracetam and topiramate to existing medications (phenobarbital, potassium bromide, and zonisamide). However, these medications were only partially effective. At 18 years of age, the patient continued to have brief focal seizures, often in clusters requiring intravenous phenobarbital for seizure control.

Whole exome sequencing was performed using a SureSelectXT Human All Exon v5 (Agilent Technologies, Santa Clara, CA), and captured libraries were sequenced using an Illumina HiSeq 2500 (Illumina, San Diego, CA) with 101 base-paired end reads. Exome data processing, variant calling, and variant annotation were performed as previously described^11^. Variant pathogenicity was predicted using SIFT, Polyphen-2, CADD and M-CAP (Table S1). Whole exome sequencing of the patient’s leukocyte-derived genomic DNA identified an *HCN1* variant: NM_021072.4, c.1195T>C, p.(Ser399Pro). Trio-based Sanger sequencing confirmed that this was a de novo variant. The variant was absent in 38 K JPN and gnomAD v2.1.1 (accessed on 22nd Feb 2023), as well as in our 408 in-house control exomes (all Japanese). The same variant was previously reported in a patient with infantile epileptic encephalopathy who had prolonged febrile seizures and intractable apneic tonic–clonic seizures starting at 4 months of age^5^. Therefore, the variant was classified as pathogenic according to the ACMG-AMP Guidelines (PS1, PS2, PM2, and PP3). The clinical and molecular genetic studies were performed in accordance with the Declaration of Helsinki and were approved by the institutional review board of Yamagata University Faculty of Medicine, Showa University School of Medicine, and Yokohama City University School of Medicine. The patient’s parents provided written informed consent.

We compared the features of the present patient with those of a previous patient carrying the same genetic variant^5^ and summarized the clinical manifestations of previously reported cases^10^ (Table 1). The present patient showed infantile-onset DEE with repetitive focal seizures, recurrent refractory status epilepticus, and progressive brain atrophy. Among previously reported patients with *HCN1* variants, brain atrophy was observed in only one patient with a p.G391D variant^5,10^. Therefore, the phenotype in the present patient appears to be more severe than that of a previous patient with the same p.S399P variant. This phenotypic variability may be caused by genetic modifiers such as a prominent family history of febrile seizures even though no other variants in the known epilepsy-related genes were identified through whole exome sequencing of this patient. Progressive brain atrophy may also have been caused by acute encephalopathy or encephalitis. However, because MRI changes were not observed during the acute phase of status epilepticus in the patient, it was unlikely that he had acute encephalopathy or encephalitis.

**Table 1:** Phenotypic manifestations of two patients with the p.S399P variant and a summary of previously reported cases.

Case	Summary by Kessi et al.^10^ (43 cases)	Previous case with p.S399P variant^5^	The present case
Sex			
Males	25/41 (61 %)	Male	Male
Females	16/41 (39 %)		
Altered protein function			
GOF	6/26 (23%)	LOF (p.Ser399Pro)	LOF (p.Ser399Pro)
LOF	10/26 (38.5%)		
Unknown	10/26 (38.5%)		
Age at seizure onset	15.4 (range, 0-84) months	4 months	2.5 months
Initial seizure semiology			
Febrile seizures	26/42 (62 %)	Febrile and afebrile TCS, generalized seizures with apnea	Hemiclonic seizures and FBTCS, prolonged for 36 hours
Tonic seizures	10/42 (24 %)		
Clonic seizures	5/42 (12 %)		
Generalized seizures	1/42 (2 %)		
Absence seizure	2/42 (5 %)		
Status epilepticus	5/9 (56 %)	No	Yes
Epilepsy syndromes	Febrile seizures, febrile seizure plus, genetic generalized epilepsy, EIEE, febrile-EIEE, EIMFS, unclassified epilepsy	EIEE	EIEE
Seizure outcome			
Seizure free	16/41 (39%)	Daily seizures	Daily seizures, occasionally in cluster
Controlled seizures	2/41 (5 %)		
Drug-resistant epilepsy	5/41 (12 %)		
Daily seizures	6/41 (15 %)		
Weekly seizures	1/41 (2 %)		
Rare seizures	3/41 (7 %)		
Monthly seizures	2/41 (5 %)		
Yearly seizures	2/41 (5 %)		
Died	4/41 (10 %)		
Unknown	2/41 (5 %)		
MRI findings	Normal 30/34 (88 %) Large cerebellum and incomplete myelination 1/34 (3 %) Mild, diffuse white matter hyperintensity 1/34 (3 %) Severe brain atrophy 1/34 (3 %) Thin corpus callosum 1/34 (3 %)	Normal	Normal at 4 months, severe brain atrophy at 8 months
Intellectual disability/ gobal developmental delay	22/43 (51 %)	Severe	Severe

*EIEE* early infantile epileptic encephalopathy, *EIMFS* epilepsy of infancy with migrating focal seizures, *FBTCS* focal to bilateral tonic-clonic seizure, *FS* febrile seizures, *GOF* gain of function, *LOF* loss of function, *MRI* magnetic resonance imaging, *TCS* tonic-clonic seizure.

Recurrent status epilepticus, refractory to intravenous anticonvulsant administration, was one of the characteristic features observed in this patient. Administration of general anesthesia was necessary to abolish a prolonged seizure lasting 36 hours. In previous studies, status epilepticus was reported in 5/18 patients with loss-of-function *HCN1* variants and in 1/13 patients with gain-of-function *HCN1* variants^9^. The p.S399P variant is also a loss-of-function variant. However, status epilepticus is caused not by a specific *HCN1* variant but by a variety of variants^9^.

Both upregulation and downregulation of HCN channels have been associated with epileptic activity in animal models^5,12^. A previous study indicated that patients with the p.G391D variant showed the most severe phenotypes among *HCN1* DEE^5^ patients, similar to the present patient with the p.S399P variant. HCN subunits have six transmembrane domains (S1–S6), including a positively charged voltage sensor (S4) and the ion-conducting pore region between S5 and S6^3^. The amino acid glycine 391, located on the intracellular interface of the S6 transmembrane domain, is adjacent to the p.S399P variant^5^. Transient expression of *HCN1* variants in Chinese hamster ovary cells showed that the protein levels of both the p.G391D and p.S399P variants were significantly decreased compared with those of wild-type proteins. In addition, whole-cell patch-clamp recordings showed no current in cells separately transfected with each variant, suggesting that these are loss-of-function variants^5^. Interestingly, when cells were cotransfected with wild-type and p.G391D variant constructs, a strong reduction in current density was observed, suggesting that p.G391D has a dominant-negative effect on heteromeric channel function. Similarly, severe DEE in the present patient may have been caused by a dominant-negative effect of the p.S399P variant.

The epileptic seizure frequency in patients with *HCN1* variants ranges from no to daily seizures, and half of patients demonstrate resistance to anti-seizure medications^5^. High-dose phenobarbital and potassium bromide were partially effective for the present patient. In murine models of p.G391D and p.M153I, administration of the sodium channel antagonists lamotrigine and phenytoin resulted in paradoxical induction of seizures; however, administration of sodium valproate did not lead to convulsive seizures^13^. In *Hcn1*^M294L^ mice carrying a homolog of the *HCN1* p.M305L variant; phenytoin, lamotrigine, retigabine, and carbamazepine increased spike frequency, whereas levetiracetam, diazepam, sodium valproate, and ethosuximide significantly reduced spike frequency^14^. Sodium channel blockers such as carbamazepine or phenytoin may aggravate seizures in Dravet syndrome with *SCN1A* variants^15^. These blockers might also aggravate seizures in patients with *HCN1* variants; therefore, early genetic diagnosis could be crucial for appropriate pharmacotherapy selection.

## HGV database

The relevant data from this Data Report are hosted at the Human Genome Variation Database at 10.6084/m9.figshare.hgv.3311.

### Supplementary information

Supplemental Table 1.

## References

[1] Biel M, Wahl-Schott C, Michalakis S, Zong X (2009). Hyperpolarization-activated cation channels: from genes to function. Physiol. Rev..

[2] Poolos, N. P. Hyperpolarization-activated cyclic nucleotide-gated (HCN) ion channelopathy in epilepsy. In: Noebels, J. L., Avoli, M., Rogawski, M. A., Olsen, R. W., & Delgado-Escueta, A. V. (eds). *Jasper’s Basic Mechanisms of the Epilepsies*, 4th edn. Oxford University Press: Oxford, USA, 85–96 (2012). 22787677

[3] Benarroch EE (2013). HCN channels: function and clinical implications. Neurology.

[4] Nava C (2014). De novo mutations in *HCN1* cause early infantile epileptic encephalopathy. Nat. Genet..

[5] Marini C (2018). *HCN1* mutation spectrum: from neonatal epileptic encephalopathy to benign generalized epilepsy and beyond. Brain.

[6] Parrini E (2017). Diagnostic targeted resequencing in 349 Patients with drug-resistant pediatric epilepsies identifies causative mutations in 30 different genes. Hum. Mutat..

[7] Bonzanni M (2018). A novel *de novo HCN1* loss-of-function mutation in genetic generalized epilepsy causing increased neuronal excitability. Neurobiol. Dis..

[8] Porro A (2021). Do the functional properties of *HCN1* mutants correlate with the clinical features in epileptic patients?. Prog. Biophys. Mol. Biol..

[9] Xie C (2022). Novel *HCN1* mutations associated with epilepsy and impacts on neuronal excitability. Front. Mol. Neurosci..

[10] Kessi M (2022). The contribution of HCN channelopathies in different epileptic syndromes, mechanisms, modulators, and potential treatment targets: a systematic review. Front. Mol. Neurosci..

[11] Saitsu H (2013). De novo mutations in the autophagy gene *WDR45* cause static encephalopathy of childhood with neurodegeneration in adulthood. Nat. Genet..

[12] Noam Y, Bernard C, Baram TZ (2011). Towards are integrated view of HCN channel role in epilepsy. Curr. Opin. Neurobiol..

[13] Merseburg A (2022). Seizures, behavioral deficits, and adverse drug responses in two new genetic mouse models of *HCN1* epileptic encephalopathy. eLife.

[14] Bleakley LE, McKenzie CE, Reid CA (2023). Efficacy of antiseizure medication in a mouse model of *HCN1* developmental and epileptic encephalopathy. Epilepsia.

[15] Wirrell EC (2022). International consensus on diagnosis and management of Dravet syndrome. Epilepsia.

