# Supplementary material for: The HCN1 p.Ser399Pro variant causes epileptic encephalopathy with super-refractory status epilepticus
Source: Hum Genome Var. 2023 Jun 23;10:20. doi: 10.1038/s41439-023-00247-8 (PMC10290089; doi:10.1038/s41439-023-00247-8)
Supplement: Supplementary file 1 — Supplemental Table 1. [file 41439_2023_247_MOESM1_ESM.doc]

**Table 1. Variant pathogenicity predicted by SIFT, Polyphen-2, CADD, and M-CAP**

| **Gene** | **Refseq** | **Variant** | **SIFT** | **PolyPhen2**  **HumVar** | **CADD**  **phred** | **M-CAP** | **GERP** | **Phast**  **Cons** | **gnomAD** | **38KJPN** |
| --- | --- | --- | --- | --- | --- | --- | --- | --- | --- | --- |
| ***HCN1*** | NM_021072.4 | c.1195T>C, p.(Ser399Pro) | 0.0 | 0.147 | 24.6 | 0.522 | 5.420 | 1.000 | - | - |
